# Lenalidomide-associated pure red cell aplasia during the treatment of POEMS syndrome: A case report and brief review of the literature

**DOI:** 10.3892/mi.2025.283

**Published:** 2025-10-29

**Authors:** Minghui Dai, Qiuling Li, Xiaohong Kuang, Jiaxun Wei, Wei Jiang, Lin Zhang, Ling Li, Yan Zou

**Affiliations:** 1 Department of Hematology, The Third Hospital of Mianyang, Sichuan Mental Health Center, Mianyang, Sichuan 621000, P.R. China; 2 Hongyuan County People's Hospital of Sichuan Province, Hongyuan, Sichuan 624400, P.R. China

**Keywords:** lenalidomide, POEMS syndrome, M-protein, pure red cell aplasia, therapy

## Abstract

Lenalidomide is widely used in the treatment of hematological malignancies, with bone marrow suppression being one of the most common hematological adverse effects associated with its use. However, lenalidomide-associated pure red cell aplasia (PRCA) remains exceptionally rare. The present study describes a case of PRCA secondary to lenalidomide therapy in a patient with POEMS syndrome. A 61-year-old woman diagnosed with POEMS syndrome received lenalidomide (25 mg/day) in combination with dexamethasone. Following 3 months of treatment, she developed severe anemia accompanied by a markedly reduced absolute reticulocyte count. Bone marrow aspiration and biopsy demonstrated a complete absence of erythroid precursors, establishing the diagnosis of PRCA. After excluding other potential causes, including viral infections, malignancies and alternative drug-related toxicities, lenalidomide was considered the most likely etiology. Lenalidomide was discontinued, and the patient was subsequently treated with cyclosporine and corticosteroids. Her anemia markedly improved after 16 weeks of therapy, with the normalization of the reticulocyte count. No recurrence was observed during 6 months of follow-up. The present study also performed a brief review of the literature, discussing this rare phenomenon.

## Introduction

As an oral immunomodulatory agent, lenalidomide has been widely employed in the treatment of hematological malignancies, including multiple myeloma, light-chain (AL) amyloidosis, lymphoma and myelodysplastic syndromes. Its principal mechanisms of action involve the inhibition of tumor cell proliferation, the enhancement of host immune responses and modulation of cytokine activity within the bone marrow microenvironment (1). pure red cell aplasia (PRCA) is characterized by the selective failure of erythropoiesis in the bone marrow. Clinically, it manifests as normocytic, normochromic anemia with a profoundly reduced reticulocyte count and a marked reduction or the absence of erythroid precursors, while granulopoiesis and megakaryopoiesis remain preserved. The etiology of PRCA is heterogeneous, encompassing viral infections, medications, autoimmune disorders and malignancies (2). The most common hematological toxicities associated with the use of lenalidomide include neutropenia, anemia and thrombocytopenia.

By contrast, lenalidomide-associated PRCA is exceedingly rare, with only 4 cases reported to date (3-5). Of these, 3 patients had MM and one had MDS. All developed acute-onset severe anemia following a period of lenalidomide therapy, and bone marrow evaluation confirmed PRCA. Each case responded favorably to cyclosporine treatment, achieving hematologic recovery. The present study describes a rare case of PRCA arising during lenalidomide therapy in a patient with POEMS syndrome.

## Case report

A 61-year-old woman was diagnosed with POEMS syndrome at the Department of Hematology, the Third Hospital of Mianyang (Sichuan, China (in May, 2021. She presented with numbness and edema of the lower extremities. Initial laboratory tests revealed the following: A white blood cell count (WBC) of 5.2x10^9^/l, a hemoglobin level of 86 g/l, a platelet count of 134x10^9^/l, and an absolute reticulocyte count (RET#) of 109.6x10^9^/l. Serum M-protein was detected at 2.88 g/l, and immunofixation electrophoresis identified a monoclonal IgA-λ band. A bone marrow smear demonstrated a mildly increased proportion of plasma cells (3%), and flow cytometry revealed 0.52% monoclonal plasma cells. An abdominal ultrasound confirmed splenomegaly, while electromyography revealed axonal injury involving the motor nerves of the lower limbs.

Following the diagnosis, the patient was initiated on the RD regimen, consisting of lenalidomide (25 mg, days 1-21) and dexamethasone (10 mg, days 1-2, 8-9, 15-16 and 22-23). Following three cycles, her lower-extremity edema and numbness improved, hemoglobin levels rose to 100 g/l and serum M-protein became undetectable. However, prior to the fourth cycle, she developed the sudden worsening of fatigue, accompanied by dizziness and palpitations. She denied fever, abdominal pain, diarrhea, nausea, vomiting, jaundice, hematuria or melena.

**Figure 1:**
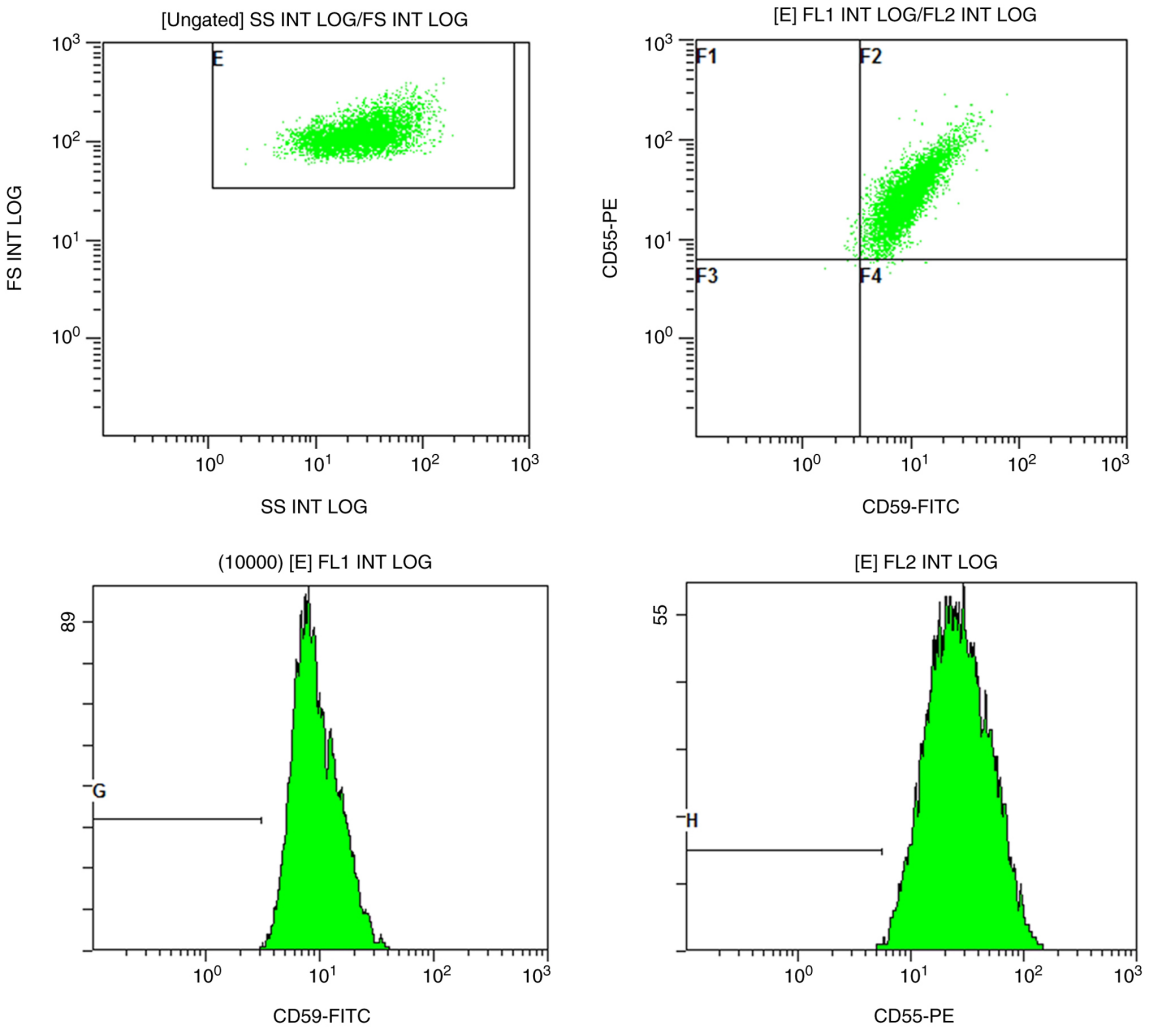
Flow cytometric analysis for paroxysmal nocturnal hemoglobinuria. Representative histograms illustrating the normal expression of CD55 and CD59 in red blood cells. No paroxysmal nocturnal hemoglobinuria clone was detected.

**Figure 2:**
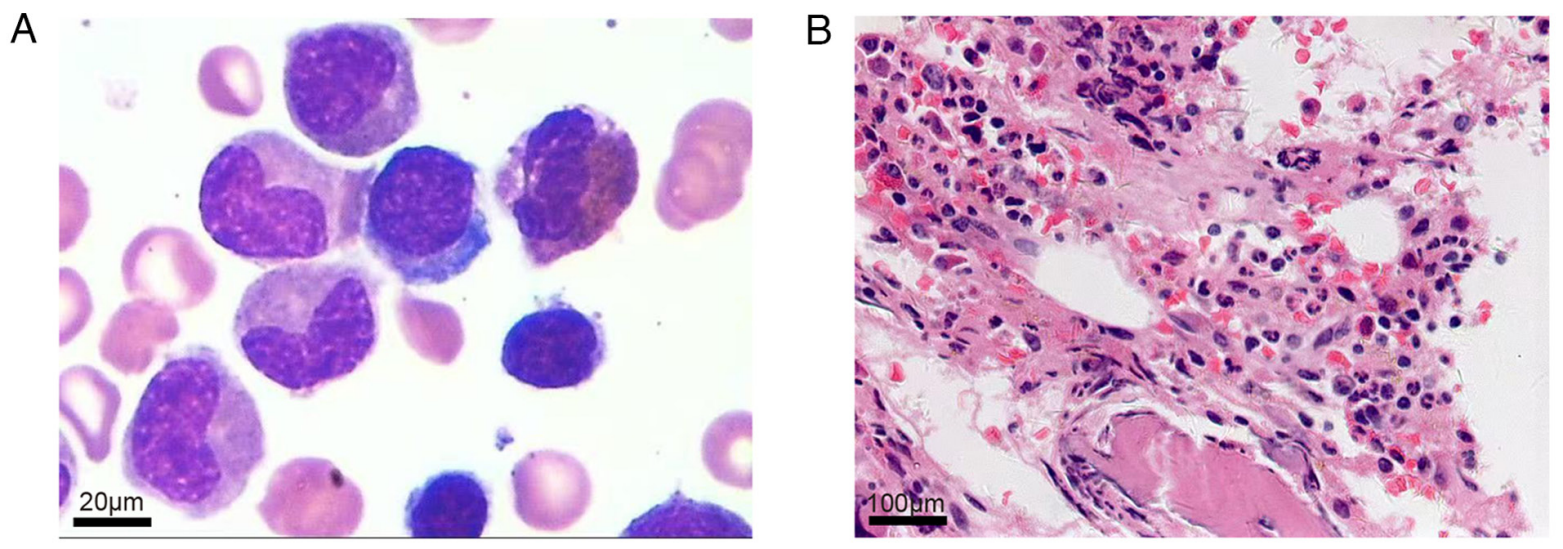
(A) Bone marrow smear findings. Hypercellular marrow illustrating 64.5% granulocytic and ~1% erythroid cells, with no evidence of blasts or dysplasia (Wright-Giemsa staining; magnification, x100; scale bar, 20 µm). (B) Bone marrow biopsy findings. Hypercellular marrow (~50%) with granulocytic predominance and markedly reduced erythropoiesis (hematoxylin and eosin staining; magnification, x40; scale bar, 100 µm).

Repeat laboratory testing revealed the following: A WBC of 6.07x10^9^/l, a hemoglobin level of 22 g/l, a platelet count of 63x10^9^/l, and a RET# of 4x10^9^/l. A hemolysis panel was negative, as was the expression of CD55 and CD59 detected using flow cytometry (the flow cytometric analysis for CD55 and CD59 expression was not outsourced to a certified third-party laboratory, KingMed Diagnostics, Guangzhou, China) (Fig. 1). The total bilirubin level was 9.6 µmol/l (reference range, 0-20 µmol/l); the autoimmune panel, including ANA, anti-dsDNA and anti-ENA antibodies was negative. Serum protein electrophoresis and immunofixation electrophoresis revealed no abnormal monoclonal bands. Serologies for hepatitis A, B, and C viruses, Epstein-Barr virus, HIV and parvovirus B19 were negative. The serum ferritin level was elevated at 2,338 ng/ml, while the levels of serum folate, vitamin B12 and vascular endothelial growth factor (VEGF) were within normal limits. A bone marrow smear revealed hypercellularity with granulocytic predominance (64.5%) and the complete absence of erythroid precursors (Fig. 2A). Fig. 2 illustrates bone marrow smear and bone marrow biopsy sections stained with Wright-Giemsa and hematoxylin and eosin (H&E), respectively. The bone marrow smear (Fig. 2A) was prepared and stained at the Department of Laboratory Medicine, The Third Hospital of Mianyang, Sichuan Mental Health Center, Sichuan, China. Bone marrow aspirate smears were air-dried and fixed in absolute methanol for 10 min at room temperature, followed by staining with Wright-Giemsa solution (Beijing Solarbio Science & Technology Co., Ltd.) for 15 min and rinsing in distilled water. The bone marrow biopsy (Fig. 2B) was processed and stained by an external laboratory, namely KingMed Diagnostics (www.kingmed.com.cn). The specimen was paraffin-embedded and sectioned at a thickness of 4 µm, fixed in 10% neutral buffered formalin for 24 h at room temperature, and stained with hematoxylin and eosin according to standard protocols. Microscopic images were captured using an Olympus BX53 light microscope (Olympus Corporation). The bone marrow biopsy revealed hypercellular marrow with marked granulocytic hyperplasia and severely reduced erythropoiesis (Fig. 2B), confirming the diagnosis of PRCA.

Lenalidomide was discontinued, and the patient was commenced on immunosuppressive therapy with cyclosporine and corticosteroids, along with supportive transfusions. Following stabilization, cyclosporine was continued on an outpatient basis, while corticosteroids were tapered according to clinical response. At the 4-month follow-up, the hemoglobin levels had increased to ~110 g/l, and serum M-protein remained undetectable.

## Discussion

PRCA is a rare form of anemia characterized by a marked reduction or complete absence of erythroid precursors in the bone marrow, leading to severe anemia. PRCA may arise from diverse etiologies, including viral infections, medications, autoimmune disorders, and malignancies. The central pathological mechanism involves immune-mediated destruction of erythroid precursor cells, encompassing both cellular and humoral immune responses (2). POEMS syndrome is an uncommon multisystem disorder associated with plasma cell dyscrasia, with an estimated incidence of <1 per million individuals (6,7). Although its exact pathogenesis remains incompletely understood, the disease is closely linked to abnormal plasma cell proliferation. These plasma cells often secrete lambda light chains and VEGF, contributing to increased vascular permeability and multisystem organ dysfunction (8-11). Other cytokines, such as interleukin (IL)-12, IL-6 and IL-1β, may also play a role in the disease process (12,13).

The occurrence of PRCA secondary to POEMS syndrome is exceedingly rare, and the literature on this association is limited. Some reports suggest that PRCA in this context may be related to abnormalities in T-cell subsets or the presence of autoantibodies, both of which can target erythroid progenitors. Furthermore, patients with POEMS syndrome often demonstrate elevated levels of VEGF, TNF-α and interferon-γ (IFN-γ), cytokines that may contribute to erythroid suppression via aberrant immune activation (7). In addition, monoclonal gammopathy (M-protein) has been implicated in the pathogenesis of PRCA. In the study by Korde *et al* (14) 24% of 51 patients with PRCA were found to harbor M-protein, and plasma cell-directed therapy led to remission in these cases. M-proteins may directly bind to erythroid precursor antigens, thereby inhibiting erythropoiesis, or activate the complement system, leading to immune-mediated destruction (14,15). Notably, PRCA associated with POEMS or M-protein typically occurs early in the disease course and is often refractory to immunosuppressive therapy. By contrast, the patient in the present study developed PRCA after achieving hematological and clinical improvement with lenalidomide. The delayed onset during disease control, coupled with the responsiveness to cyclosporine and corticosteroids, argues against POEMS- or M-protein-related PRCA and supports a drug-related etiology. Nevertheless, given the established associations between PRCA and plasma cell dyscrasias, a causal role of POEMS or M-protein cannot be completely excluded. The temporal sequence, however, suggests that lenalidomide-induced PRCA is the most plausible explanation in this case.

Lenalidomide is an oral immunomodulatory agent widely used in hematological malignancies. Its primary mechanisms of action include the inhibition of tumor cell proliferation, the enhancement of host immune responses, and the modulation of cytokines such as VEGF and TNF-α. It has also demonstrated efficacy in POEMS syndrome (1,16). Hematological toxicities, however, are common, most notably neutropenia, anemia and thrombocytopenia. PRCA is a rare, yet increasingly recognized adverse effect of lenalidomide. To date, only 4 cases have been reported (Table I): Of these, 3 patients had multiple myeloma and 1 patient had myelodysplastic syndrome. Consistent with the case in the present study, all cases exhibited abrupt severe anemia following exposure to lenalidomide, the analysis of bone marrow revealed the absence of erythropoiesis, and favorable responses to cyclosporine therapy. The exact mechanisms of lenalidomide-associated PRCA remain unclear. Several hypotheses have been proposed: i) Immunomodulatory effects: Lenalidomide enhances T-cell and natural killer cell activity. While beneficial for antitumor immunity, this may lead to the aberrant targeting of erythroid progenitors. ii) Cytokine dysregulation: Lenalidomide alters the expression of cytokines, notably IL-2 and IFN-γ, which are known inhibitors of erythropoiesis (1). iii) MHC class I upregulation: Recently, Hu *et al* (5) proposed a novel mechanism, demonstrating that lenalidomide upregulates MHC-I expression on erythroid precursors, thereby enhancing CD8^+^ T-cell recognition and cytotoxicity. This provides theoretical support for a ‘drug-immune cross-talk’ model. However, the evidence is preliminary: Their study involved an in-depth analysis of only 1 case with an additional validation, without larger cohorts or controls, precluding estimation of incidence or relative risk. Moreover, erythroid cells from healthy donors did not exhibit this phenotype, suggesting that individual susceptibility is required (5). Thus, while biologically plausible, these mechanisms remain speculative and require confirmation in larger cohorts. Therefore, PRCA induced by lenalidomide is likely the result of a multifactorial immunologic process. Beyond lenalidomide, other immunomodulatory drugs (IMiDs), such as thalidomide and pomalidomide, also exert profound immunomodulatory and anti-angiogenic effects (17,18). Reports of severe cytopenias, including anemia and aplastic presentations, exist with these agents, suggesting that the risk of profound erythroid suppression may not be unique to lenalidomide. This raises the possibility of a broader class effect among IMiDs, although direct PRCA cases remain rare.

In the case in the present study, the patient developed severe anemia following lenalidomide treatment. Laboratory tests revealed a markedly reduced reticulocyte count, and bone marrow examination demonstrated a profound decrease in erythroid precursors with preserved granulopoiesis and megakaryopoiesis, consistent with PRCA. Other common causes of PRCA were excluded, making lenalidomide the most likely etiological factor. Following the discontinuation of lenalidomide and initiation of cyclosporine combined with corticosteroids, the patient's condition stabilized, and hemoglobin levels gradually returned to normal.

When PRCA arises in the setting of POEMS syndrome or other M protein-related disorders, it is essential to determine whether the anemia is secondary to the underlying disease. In such cases, therapy should primarily target the primary disorder. By contrast, lenalidomide-associated PRCA requires a different approach. Recommended strategies include the withdrawal of lenalidomide, supportive care and the initiation of immunosuppressive therapy. While some patients may achieve spontaneous recovery after discontinuing the drug, those with persistent or severe anemia should receive early immunosuppressive treatment along with transfusion support. For the underlying disease, an alternative regimen should be selected to avoid disease progression.

In conclusion, the present case report highlights a rare, yet clinically significant complication of lenalidomide therapy. The pathogenesis appears to involve complex immunologic processes; however, current evidence remains preliminary. While lenalidomide-induced PRCA is the most likely explanation in the patient described herein, the possibility of contribution from POEMS or M-protein-related mechanisms cannot be completely excluded. Larger studies incorporating genetic and immunologic profiling are warranted to clarify predisposition factors, confirm causality and guide safer therapeutic strategies.

## Figures and Tables

**Table I:** Clinical characteristics of the four cases of pure red cell aplasia identified in the literature.

Patient no.	First author, year of publication	Sex/age (years)	Underlying disease	Clinical presentation	Treatment regimen	Outcome	(Refs.)
1	Ito, 2018	F/77	MM	Anemia; erythroid precursors 0.4%	Cyclosporine	Hemoglobin increased from 68 to 102 g/l after 1 month	(3)
2	Ito, 2018	M/73	MM	Anemia; erythroid precursors 3.1%	Cyclosporine	Hemoglobin increased from 79 to 99 g/l after 1 month; died of underlying disease progression at 6 months	(3)
3	Dolai, 2014	M/47	MDS	Anemia; erythroid precursors <5%	Cyclosporine + prednisone	Hemoglobin returned to pre-PRCA levels after 2 months	(4)
4	Hu, 2024	-	MM	Anemia; erythroid precursors absent	Cyclosporine	Hemoglobin increased from 55 to 109 g/l after 5 months	(5)

## Data Availability

The data generated in the present study may be requested from the corresponding author.

## References

[1] Chanan-Khan AA, Cheson BD (2008). Lenalidomide for the treatment of B-cell malignancies. J Clin Oncol.

[2] Gurnari C, Maciejewski JP (2021). How I manage acquired pure red cell aplasia in adults. Blood.

[3] Ito T, Nakaya A, Fujita S, Satake A, Nakanishi T, Azuma Y, Tsubokura Y, Konishi A, Hotta M, Yoshimura H (2018). Secondary pure red cell aplasia in multiple myeloma treated with lenalidomide. Leuk Res Rep.

[4] Dolai TK, Dutta S, Mandal PK, Saha S, Bhattacharyya M (2014). Lenalidomide-induced pure red cell aplasia. Turk J Haematol.

[5] Hu Q, Liu Y, Yue Q, Zhou S, Jin X, Lin F, Huang XJ, Zhuang J, Lu J, Gao X, Lee HY (2024). Lenalidomide-induced pure red cell aplasia is associated with elevated expression of MHC-I molecules on erythrocytes. Nat Commun.

[6] Li J, Zhou DB (2013). New advances in the diagnosis and treatment of POEMS syndrome. Br J Haematol.

[7] Dispenzieri A (2023). POEMS syndrome: Update on diagnosis, risk-stratification, and management. Am J Hematol.

[8] Nishi J, Arimura K, Utsunomiya A, Yonezawa S, Kawakami K, Maeno N, Ijichi O, Ikarimoto N, Nakata M, Kitajima I (1999). Expression of vascular endothelial growth factor in sera and lymph nodes of the plasma cell type of Castleman's disease. Br J Haematol.

[9] Mineta M, Hatori M, Sano H, Hosaka M, Kokubun S, Hiroki E, Hatakeyama A, Ogasawara T (2006). Recurrent Crow-Fukase syndrome associated with increased serum levels of vascular endothelial growth factor: A case report and review of the literature. Tohoku J Exp Med.

[10] Kanai K, Kuwabara S, Misawa S, Hattori T (2007). Failure of treatment with anti-VEGF monoclonal antibody for long-standing POEMS syndrome. Intern Med.

[11] Sekiguchi Y, Misawa S, Shibuya K, Nasu S, Mitsuma S, Iwai Y, Beppu M, Sawai S, Ito S, Hirano S (2013). Ambiguous effects of anti-VEGF monoclonal antibody (bevacizumab) for POEMS syndrome. J Neurol Neurosurg Psychiatry.

[12] Soubrier M, Dubost JJ, Serre AF, Ristori JM, Sauvezie B, Cathebras P, Piette JC, Chapman A, Authier FJ, Gherardi RK (1997). Growth factors in POEMS syndrome: Evidence for a marked increase in circulating vascular endothelial growth factor. Arthritis Rheum.

[13] Kanai K, Sawai S, Sogawa K, Mori M, Misawa S, Shibuya K, Isose S, Fujimaki Y, Noto Y, Sekiguchi Y (2012). Markedly upregulated serum interleukin-12 as a novel biomarker in POEMS syndrome. Neurology.

[14] Korde N, Zhang Y, Loeliger K, Poon A, Simakova O, Zingone A, Costello R, Childs R, Noel P, Silver S (2016). Monoclonal gammopathy-associated pure red cell aplasia. Br J Haematol.

[15] Verma R (2020). Molecular pathways engaged by immunomodulatory agents in monoclonal gammopathy-associated pure red cell aplasia rescue. Front Oncol.

[16] Li J, Huang XF, Cai QQ, Wang C, Cai H, Zhao H, Zhang L, Cao XX, Gale RP, Zhou DB (2018). A prospective phase II study of low dose lenalidomide plus dexamethasone in patients with newly diagnosed polyneuropathy, organomegaly, endocrinopathy, monoclonal gammopathy, and skin changes (POEMS) syndrome. Am J Hematol.

[17] Franks ME, Macpherson GR, Figg WD (2004). Thalidomide. Lancet.

[18] Lacy MQ, McCurdy AR (2013). Pomalidomide. Blood.

